# Seahorse Attenuated DSS‐Induced Depression in Mice by Inhibiting Neuroinflammation and Ferroptosis

**DOI:** 10.1002/fsn3.70482

**Published:** 2025-06-22

**Authors:** Pei‐Lu Chen, Ming Li, Xin‐Yu Wang, Xian‐Zhu Qiu, Feng‐Yan Qiu, Le‐Yun Zheng, Li‐Tao Yi, Jia‐Yuan Zhang, Guang‐Hui Xu

**Affiliations:** ^1^ Xiamen Medicine Research Institute Xiamen Fujian Province China; ^2^ College of Pharmacy Fujian University of Traditional Chinese Medicine Fuzhou Fujian China; ^3^ Xiamen Health and Medical Big Data Center Xiamen Fujian Province China; ^4^ Department of Chemical and Pharmaceutical Engineering College of Chemical Engineering, Huaqiao University Xiamen Fujian Province China; ^5^ Xiamen Anz Health Co., Ltd Xiamen Fujian Province China; ^6^ Xiamen Ocean Vocational College Xiamen Fujian Province China; ^7^ Xiamen Key Laboratory of Natural Medicine Research and Development Xiamen Fujian Province China

**Keywords:** depression, ferroptosis, neuroinflammation, seahorse

## Abstract

Seahorse ( *Hippocampus abdominalis* ), a small fish, has been extensively utilized in traditional Chinese medicine to enhance and harmonize vital energy throughout the body and brain. This study aimed to elucidate the therapeutic role and underlying mechanism of seahorse in treating depressive symptoms. The therapeutic potential of seahorse was investigated in mice induced by dextran sulfate sodium (DSS) via behavioral tests, histopathological examinations, immunofluorescence staining, and transmission electron microscopy detection. Our findings revealed that seahorse effectively alleviated colitis symptoms by DSS, as shown by reduced inflammatory markers and enhanced expression of claudin‐1 in the colonic tissues. More importantly, these gastrointestinal improvements were paralleled by significant attenuation of depressive behaviors, including improved anhedonia and reduced despair‐like responses. Furthermore, seahorse exhibited a potent anti‐inflammatory effect on brain tissues, evidenced by a decreased number of microglia in the hippocampal CA1 region, reduced expression of pNF‐κB and NLRP3, and lowered cytokine levels. Additionally, seahorse promoted neurogenesis in the hippocampal DG region, enhanced brain‐derived neurotrophic factor (BDNF) content in the CA1 region, and induced the pNrf2‐mediated expression of HO‐1, GPX4, and SLC7A11, collectively counteracting DSS‐induced hippocampal ferroptosis. Transmission electron microscopy of mitochondria further confirmed that seahorse ameliorated DSS‐induced mitochondrial atrophy and cristae deficiency. These results demonstrated that seahorse reversed IBD and comorbid depressive symptoms by regulating inflammation and ferroptosis. Our study highlights the multifaceted efficacy of seahorse in alleviating IBD and comorbid depressive symptoms, potentially offering a novel therapeutic avenue for these conditions.

## Graphical abstract

This study found through animal experiments that the *Hippocampus abdominalis* can effectively alleviate the symptoms of DSS‐induced ulcerative colitis and depressive‐like behaviors, mainly by reducing inflammatory cytokine levels, promoting neurogenesis, enhancing the expression of brain‐derived neurotrophic factor (BDNF), and reducing DSS‐induced mitochondrial damage.

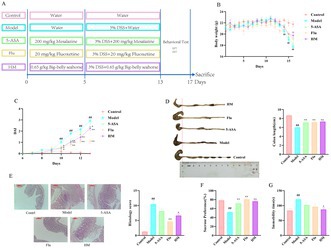

## 1 Introduction

Depression is a multifaceted psychiatric disorder characterized by persistent low mood, anhedonia, and cognitive and somatic impairment. According to the World Health Organization, depression is projected to become the leading cause of disability globally by 2030 (Chan et al. 2024). Although pharmacological treatments exist, limitations including incomplete response rates, treatment resistance, and adverse effects necessitate the exploration of novel therapeutic options. Growing evidence underscores the critical relationship between systemic inflammation and neuropsychiatric disorders, particularly through gut–brain axis mechanisms in depression (Clapp et al. 2017; Ouabbou et al. 2020).

Dextran sulfate sodium (DSS)‐induced colitis has become a standard experimental model for studying inflammatory bowel diseases (IBD) (Chassaing et al. 2014). Emerging research indicates that DSS‐induced gut inflammation can extend to the central nervous system, leading to depressive‐like behaviors in animal models (Dempsey et al. 2019; Zhao et al. 2022). These behavioral changes are mediated through gut dysbiosis, elevated pro‐inflammatory cytokine production, and disrupted neurochemical signaling, all of which collectively impair brain function (Réus et al. 2023). Notably, pro‐inflammatory cytokines including tumor necrosis factor‐alpha (TNF‐α) and interleukin‐6 (IL‐6)—which are upregulated in both colonic and brain tissues during DSS‐induced colitis—contribute to depressive symptoms by disrupting the hypothalamic–pituitary–adrenal (HPA) axis and impairing neuroplasticity (Ma et al. 2022; Puppala et al. 2023).

Ferroptosis, an iron‐dependent, lipid peroxidation‐driven form of regulated cell death (Yang and Stockwell 2016), has recently been implicated in depression pathophysiology (Chu et al. 2023). This process disrupts mitochondrial function and amplifies oxidative stress, both of which are exacerbated by systemic inflammation. The hippocampus—a key region for mood regulation—shows particular susceptibility to these pathological changes, underscoring the therapeutic potential of agents that simultaneously target inflammatory and ferroptotic pathways.

Seahorse ( *Hippocampus abdominalis* ), a marine species widely used in traditional Chinese medicine, has demonstrated general antidepressant and anti‐inflammatory properties in previous studies (Elbandy 2022; Kang et al. 2017). However, its role in modulating ferroptosis—a critical mechanism bridging oxidative stress and neuroinflammation in depression—had not been investigated until now. Although Kang et al. (2017) established the antidepressant effects of seahorse through antioxidative pathways (Kang et al. 2017), their work did not examine its influence on key ferroptosis‐related proteins (e.g., GPX4, SLC7A11) or neuroinflammatory markers (e.g., NLRP3, pNF‐κB) in the hippocampus. Our study breaks new ground by demonstrating the dual therapeutic capacity of seahorse to concurrently mitigate neuroinflammation and ferroptosis in a DSS‐induced depression model.

This study investigated the antidepressant‐like effects of seahorse extract in a DSS‐induced depression mouse model, specifically examining its dual modulation of inflammatory and ferroptotic pathways. We tested the hypothesis that seahorse extract attenuates depressive‐like behaviors through concurrent regulation of hippocampal inflammation (via TLR4/NF‐κB/NLRP3 signaling) and ferroptosis (via GPX4/SLC7A11 pathways). Our comprehensive approach evaluated both neuroimmune markers and ferroptosis‐related proteins to establish the therapeutic potential of seahorse for IBD‐associated depression.

## 2 Materials and Methods

### 2.1 Materials and Chemicals

Seahorse ( *Hippocampus abdominalis* ) was purchased from Xiamen Xiaodeng Aquatic Technology Co. Ltd. (Xiamen, China), which was identified by Cheng‐Fu Li (Xiamen Hospital of Traditional Chinese Medicine). DSS (molecular weight 40 kDa) and fluoxetine were purchased from Maclin (Shanghai, China). Mesalazine was purchased from Sunflower Pharmaceutical Group (Jiamusi, China).

### 2.2 HPLC Analysis

Seahorse (Hippocampus abdominalis) samples were washed, dried to maintain moisture content below 6%, and sequentially processed through coarse grinding (10 mm particles using a pulverizer), fine grinding (80‐mesh sieve passage), and cryogenic pulverization (−10°C for 50 min using a wall‐breaking ultra‐micro pulverizer) to ensure uniform particle size. For amino acid analysis, samples were hydrolyzed with 10 mL 6 M HCl at 150°C for 1 h, filtered, and evaporated to dryness at 100°C. The residue was reconstituted in 50 mL 0.1 M HCl, with 5 mL aliquots derivatized using 5 mL 0.1 M HCl, 3 mL 0.1 M phenylisothiocyanate‐acetonitrile, and 3 mL 1 M triethylamine‐acetonitrile (1 h incubation at room temperature, light‐protected). After adding 25 mL 50% acetonitrile, phase separation was achieved by *n*‐hexane extraction (10 mL, 10 min standing), with the aqueous phase filtered (0.45 μm) for HPLC analysis using an Ultimate Amino Acid C18 column (5 μm, 4.6 × 250 mm) with gradient elution (0.1 M sodium acetate‐acetonitrile [93:7, eluent A] vs. 80% acetonitrile [eluent B] at 0–11 min:100% A; 11–13.9 min:93% A; 13.9–14 min:88% A; 14–29 min:85% A; 29–32 min:66% A; 32–35 min:30% A; 35–42 min:0% A; 42–45 min:0% A; 45–60 min:100% A).

### 2.3 Animals and Experimental Design

Male C57BL/6 mice (6 weeks old, 16–20 g) were procured from Shanghai Slac Laboratory Animal Co. Ltd. (Shanghai, China) and housed under standardized conditions (22°C ± 1°C, 12 h light/dark cycle) with ad libitum access to autoclaved water and standard chow. Housing parameters included: 5 mice/cage (polycarbonate cages, 30 × 20 × 15 cm), corn cob bedding (autoclaved, changed twice weekly), and nesting material for enrichment. After a 7‐day acclimation period, mice were randomly assigned to groups (*n* = 10/group) via computer‐generated sequences, with sample size determined by power analysis (*α* = 0.05, power = 0.8, effect size = 1.5 based on prior DSS studies). All procedures complied with international animal research standards and were approved by Huaqiao University (A2022043) in strict adherence to China Council on Animal Care guidelines and the 3Rs principles (Replacement, Reduction, and Refinement).

**FIGURE 1:**
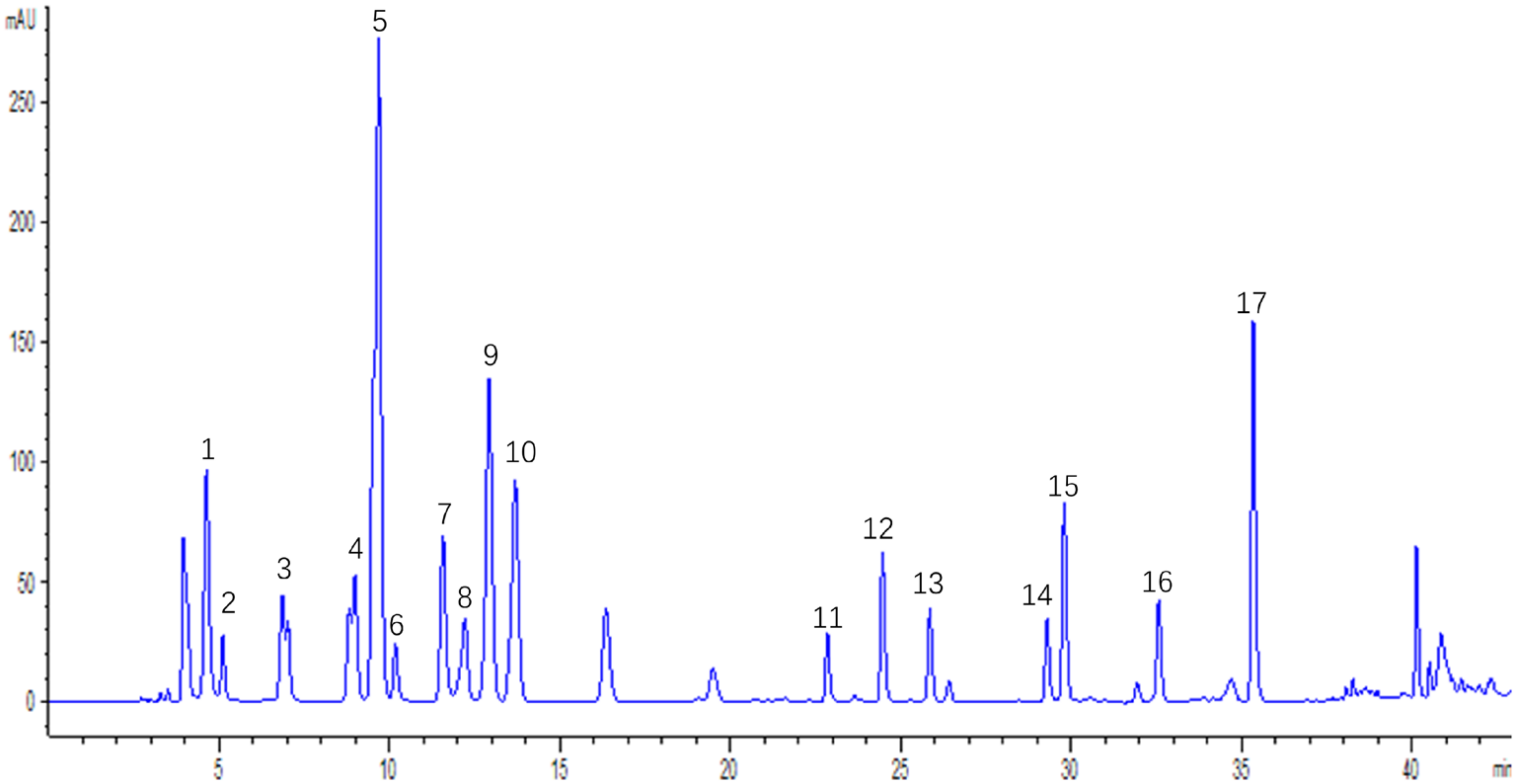
HPLC profile of seahorse at 254 nm, 17 peaks were elucidated: 1: Aspartic acid; 2: Glutamate; 3: Hydroxyproline; 4: Serine; 5: Glycine; 6: Histidine; 7: Arginine; 8: Threonine; 9: Alanine; 10: Proline; 11: Tyrosine; 12: Valine; 13: Methionine; 14: Isoleucine; 15: Leucine; 16: Phenylalanine; 17: Lysine.

Following a 7‐day acclimation period, mice were randomly allocated into five experimental groups (*n* = 10/group) using a computer‐generated sequence: (1) normal control (untreated), (2) DSS model (3% DSS in drinking water), (3) positive control 1 (mesalazine 200 mg/kg), (4) positive control 2 (fluoxetine 20 mg/kg), and (5) seahorse treatment (0.65 g/kg). The seahorse dosage was determined through preliminary dose–response studies (data not shown). The complete experimental timeline and treatment protocol are illustrated in Figure 1A.

### 2.4 Disease Activity Index (DAI)

Colitis severity was assessed daily using three clinical parameters: body weight loss, stool consistency, and hematochezia. These indicators were quantified using the Disease Activity Index (DAI) scoring system, following established criteria (Wang et al. 2024). The composite DAI scores, which integrate all three clinical measures, are presented in Table 1.

**TABLE 1:** Disease activity index.

Score	0	1	2	3	4
Fecal character	Normal	Loose	Loose	Diarrhea	Diarrhea
Gross bleeding	Negative	Negative	Hemoccult positive	Hemoccult positive	Gross bleeding
Weight loss%	0	1–5	5–10	10–15	> 15

### 2.5 Sucrose Preference Test

The sucrose preference test was performed to assess anhedonia, following established protocols (Zhang et al. 2022). Mice were first acclimated to a 1% (w/v) sucrose solution for 24 h with free access to both sucrose and water bottles. After 12 h food/water deprivation, mice were presented with two pre‐weighed bottles (1% sucrose vs. water) for 24 h. Sucrose preference was calculated as: [sucrose intake/(sucrose + water intake)] × 100%, with bottle weights recorded at 0 and 24 h.

### 2.6 Forced Swimming Test

The forced swim test was conducted as previously described (Cui et al. 2023). Mice were individually placed in a transparent cylindrical tank (15‐cm water depth, 24°C ± 1°C) for 6 min. After a 2 min habituation period, immobility time (defined as passive floating with minimal movements) was recorded during the final 4 min.

### 2.7 Histological Evaluation

Following euthanasia, colons were excised, measured for length, and photographed. For histology, colon segments were fixed in 4% paraformaldehyde (24 h, 4°C), paraffin‐embedded, and sectioned (4 μm) using a microtome. Sections were H&E‐stained and scored for pathological changes (inflammatory infiltration, crypt damage, epithelial integrity) according to established criteria (Zhang et al. 2023), with results presented in Table 2.

**TABLE 2:** Pathology score.

Score	0	1	2	3	4
1. Severity of inflammation	No or rare inflammatory cells in lamina propria	Increased inflammatory cells in lamina propria	Extension of inflammatory cells to the submucosa	Downward infiltration of inflammatory cells through the lamina propria	
2. Degree of damage	No mucosal damage	Epithelial mucosal discrete lesions	Mucosal erosions or ulcers	Lesions breaking through the lamina propria downward infiltration	
3. Injury of crypt	No damage	Less than 5% affected	Less than 33% damage	Less than 66% damage	Complete disappearance of crypt and epithelium
4. Percentage of the area involved		1%–25%	26%–50%	51%–75%	76%–100%

### 2.8 Transmission Electron Microscope (TEM) Analysis

Following established protocols (Zhou et al. 2025), hippocampal tissues were immediately excised posteuthanasia and sectioned into 1 mm^3^ pieces. Tissues were primarily fixed in pre‐chilled 2.5% glutaraldehyde (in 0.1 M phosphate buffer, pH 7.4) for 24 h at 4°C, followed by secondary fixation in 1% osmium tetroxide (in 0.1 M PBS) for 2 h at room temperature, with three subsequent PBS washes (15 min each). Dehydration was performed through a graded ethanol series (30%, 50%, 70%, 80%, 95%, and 100%, 10 min per concentration). Samples were then infiltrated first in a 1:3 mixture of epoxy resin (EMbed‐812) to acetone (1 h at 37°C), then 1:1 resin:acetone (3 h at 37°C), followed by pure resin overnight at 37°C. After embedding in fresh resin and polymerization (60°C, 48 h), ultrathin sections (80 nm) were cut using a Leica UC7 ultramicrotome with a diamond knife, collected on 200‐mesh copper grids, and double‐stained with 2% uranyl acetate in 50% ethanol (10 min) and Reynolds' lead citrate (10 min). Grids were examined using a Hitachi H7650 TEM at 80 kV, with digital images captured. Mitochondrial dimensions were quantified using Image Pro Plus 6.0 after calibration. The size of mitochondria was measured using Image Pro Plus. In brief, the ruler was first calibrated (cm as unit, *x* = 0.1/*y* = 0.1 as pixel, 1 as scaling), then the size of the mitochondria was measured.

### 2.9 Immunofluorescence Staining

Immunofluorescence staining was performed as previously described (Zhang et al. 2022). Briefly, brain and colon tissues were fixed in 4% paraformaldehyde (24 h, 4°C) and cryoprotected through graded sucrose solutions (10%, 20%, and 30% in PBS, 24 h each) before OCT embedding. Coronal brain sections (18 μm) and colon sections (15 μm) were cut using a cryostat (Leica CM1950), collected on Superfrost Plus slides, and stored at −20°C until use. For staining, sections were equilibrated to room temperature (10 min), postfixed with immunostaining fixative (10 min, RT), and washed with TBST (3 × 5 min). Antigen retrieval was performed using citrate buffer (pH 6.0, 5 min, RT) followed by TBST washes (2 × 3 min). After blocking with 5% normal goat serum in TBST (1 h, RT), sections were incubated with primary antibodies (diluted in blocking solution, 4°C overnight), washed (TBST 3 × 5 min), and incubated with species‐matched Alexa Fluor‐conjugated secondary antibodies (1:500, 3 h, RT). Nuclei were counterstained with DAPI (1 μg/mL, 5 min), and slides were coverslipped using Fluoromount‐G. Imaging was performed on a Leica TCS SP8 confocal microscope with LAS X software, maintaining identical acquisition settings across compared groups.

Primary antibodies were against the following proteins: NF‐kB p65 (1:200; ab86299, Abcam), AIF (1:200; PA5143222, Thermo Fisher), IL‐6 (1:200; ab9324, Abcam), NLRP3 (1:200; ab263899, Abcam), TNF‐α (1:200; AF‐A10‐SP, R&D), GPX4 (1:200; NBP3‐07344, Novus Biologicals), BDNF (1:200; CST47808, Cell Signaling Technology), SLC7A11 (1:200; MA5‐44922, Thermo Fisher), Neun (1:200; ab279297, Abcam), DCX (1:200; CST47808, Cell Signaling Technology), Hexokinase (1:200; NBP1‐51644, Novus Biologicals), IBA1 (1:200; ab5076, Abcam), pNrf2 (1:200; PA5‐67520, Thermo Fisher), IL‐1β (1:200; cat log no. AF‐401‐SP, R&D), Claudin‐1 (1:200; PA5‐32350, Thermo Fisher), TLR4 (1:200; MAB2759‐SP, R&D).

### 2.10 Statistical Analyses

All experimental data are presented as mean ± standard error of the mean (SEM). Intergroup comparisons were analyzed using one‐way analysis of variance (ANOVA) with Tukey's post hoc test for multiple comparisons, implemented in GraphPad Prism 9.0. Statistical significance was set at *p* < 0.05 for all analyses. Data visualization was performed using GraphPad Prism's built‐in graphing tools, with consistent formatting applied across all figures.

## 3 Results

### 3.1 Seahorse HPLC Analysis

HPLC was utilized to analyze the amino acid composition of seahorses, revealing a diverse profile consisting of 17 primary amino acids. By comparing the retention times of peaks observed in the HPLC analysis with those of standard amino acid solutions, we identified several key amino acids present in seahorse. Notably, the analysis indicated the presence of aspartic acid, glutamate, hydroxyproline, serine, glycine, among others. These findings were graphically represented in Figure 1, where each peak corresponded to a specific amino acid, identified based on its unique retention time. This detailed analysis provided insight into the amino acid composition of seahorses, indicating their potential nutritional and therapeutic value, given the importance of amino acids in various biological processes.

### 3.2 Seahorse Reversed the Both Colitis and Depression Symptoms in DSS‐Induced Mice

**FIGURE 2:**
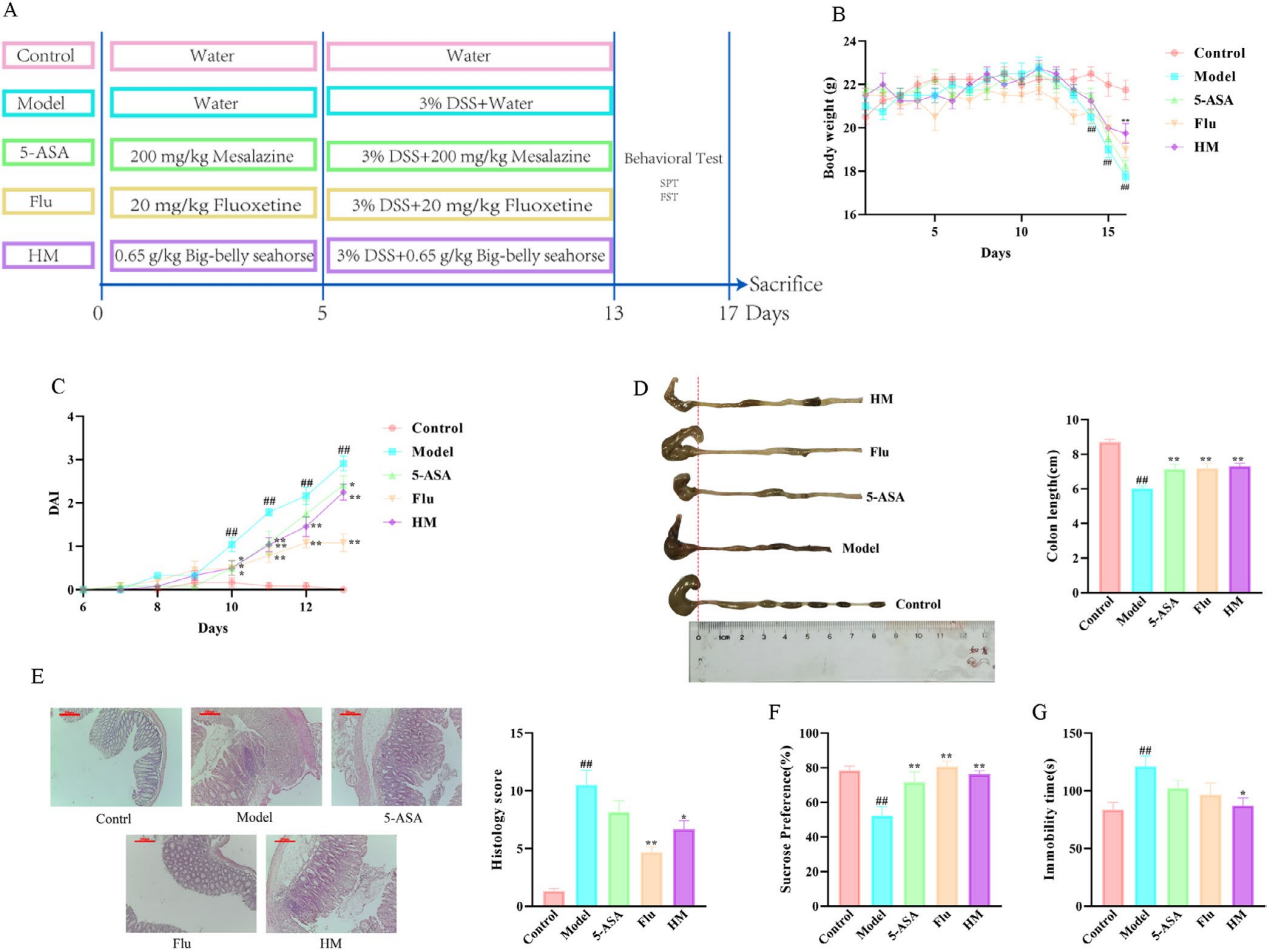
Effects of seahorse in ameliorating colitis and depression symptoms in DSS‐induced mice (*n* = 10). (A) Schematic representation of the experimental design. (B) Changes in body weight. (C) DAI scores. (D) Colon length. (E) Representative histological images of colon sections from each group stained with H&E; pathology scores (right panel) were evaluated using the criteria in Table 2: severity of inflammation (0–4), degree of damage (0–4), crypt damage (0–4), and percentage of affected area (0–4). Scale bars represent 200 μm. (F) Sucrose preference. (G) Immobility time. #*p* < 0.05 and ##*p* < 0.01 vs. control group; *p < 0.05 and **p < 0.01 vs. DSS‐induced model group.

To assess the impact of seahorse extract on depression‐like behaviors in a DSS‐induced colitis model, mice were administered seahorse extract concurrently with DSS treatment, as detailed in Figure 2A. A progressive loss of body weight was observed beginning on the sixth day after modeling (Figure 2B). Diarrhea onset was evident by the third day, with visible blood in the stool appearing by the fourth day of DSS administration. Both diarrhea and bloody stools intensified with continued DSS treatment. However, seahorse extract administration significantly improved the DAI score compared to the DSS‐only group (Figure 2C), indicating effective attenuation of DSS‐induced symptoms including weight loss, diarrhea, and fecal bleeding.

Following treatment, colonic specimens were collected for analysis. Colon length measurements and photographic documentation showed that seahorse extract notably counteracted DSS‐induced colon shortening (Figure 2D). Histopathological examination using H&E staining (Figure 2E) revealed well‐preserved colonic architecture in control groups, characterized by densely arranged intestinal glands and abundant goblet cells. In contrast, DSS‐treated groups exhibited severe disruption of intestinal glandular structure, extensive inflammatory cell infiltration in the basal layer, significant goblet cell loss, and marked mucosal wall thickening. The 5‐ASA treatment group showed moderate improvement in colonic injury with slightly reduced mucosal thickening, though inflammatory infiltration and partial glandular destruction remained evident. The fluoxetine group demonstrated substantial improvements, with structurally intact colonic mucosa, restored goblet cell populations, minimal inflammatory infiltration, and reduced mucosal thickening. Similarly, seahorse extract treatment produced significant improvements, including reduced mucosal thickening and inflammatory cell infiltration compared to the DSS model group, along with better preserved glandular structure and increased goblet cell numbers.

Behavioral assessments, including the sucrose preference test and forced swim test (Figure 2F,G), showed that seahorse extract administration effectively reversed the DSS‐induced reduction in sucrose preference, indicating amelioration of anhedonia. Additionally, seahorse extract significantly decreased immobility time in DSS‐treated mice. These findings collectively demonstrated that seahorse extract can effectively reverse depression‐like behaviors following DSS‐induced ulcerative colitis.

### 3.3 Seahorse Alleviated the Impaired Tight Junction Protein Claudin‐1 in Colon

**FIGURE 3:**
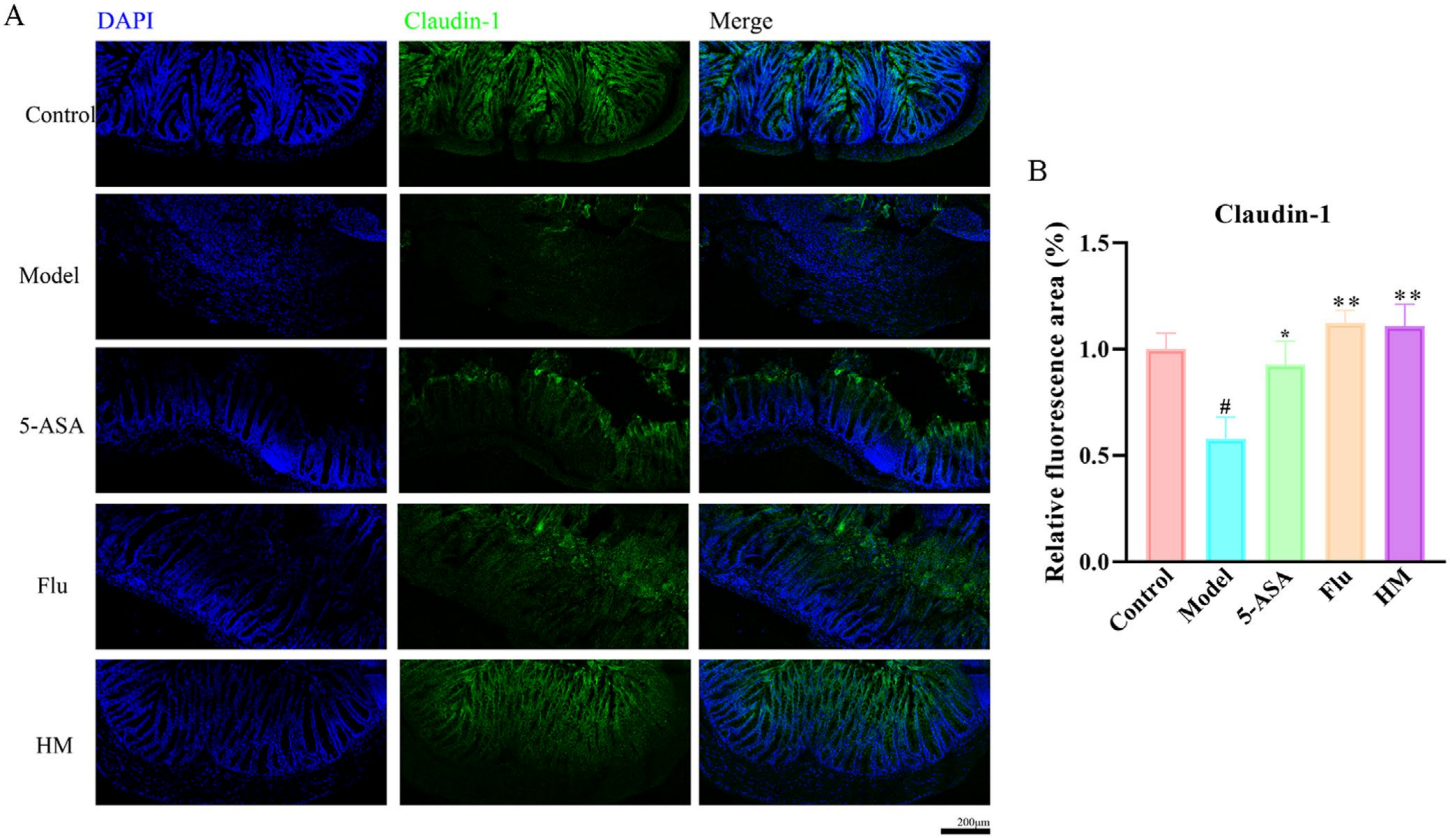
Seahorse alleviated the impaired tight junction protein claudin‐1 in DSS‐induced mice (*n* = 8). (A) Immunofluorescence staining for claudin‐1 in colonic sections of mice. (B) The histogram of claudin‐1 relative intensity. Scale bars represent 200 μm. #*p* < 0.05 vs. control group; *p < 0.05 and **p < 0.01 vs. DSS‐induced model group.

We next investigated the effects of seahorse extract on key tight junction proteins in colonic tissues, as DSS compromises the intestinal barrier and promotes leakage of toxic intestinal chemicals (Wang et al. 2024; Yuan et al. 2024). Our results demonstrated that seahorse extract treatment significantly enhanced claudin‐1 expression in colonic tissues of DSS‐treated mice compared to untreated DSS controls (Figure 3). This restoration of claudin‐1 levels suggested that seahorse extract might help repair the damaged intestinal barrier in colitis.

### 3.4 Seahorse Inhibited the Inflammation in the Colonic Tissue of DSS‐Induced Mice

**FIGURE 4:**
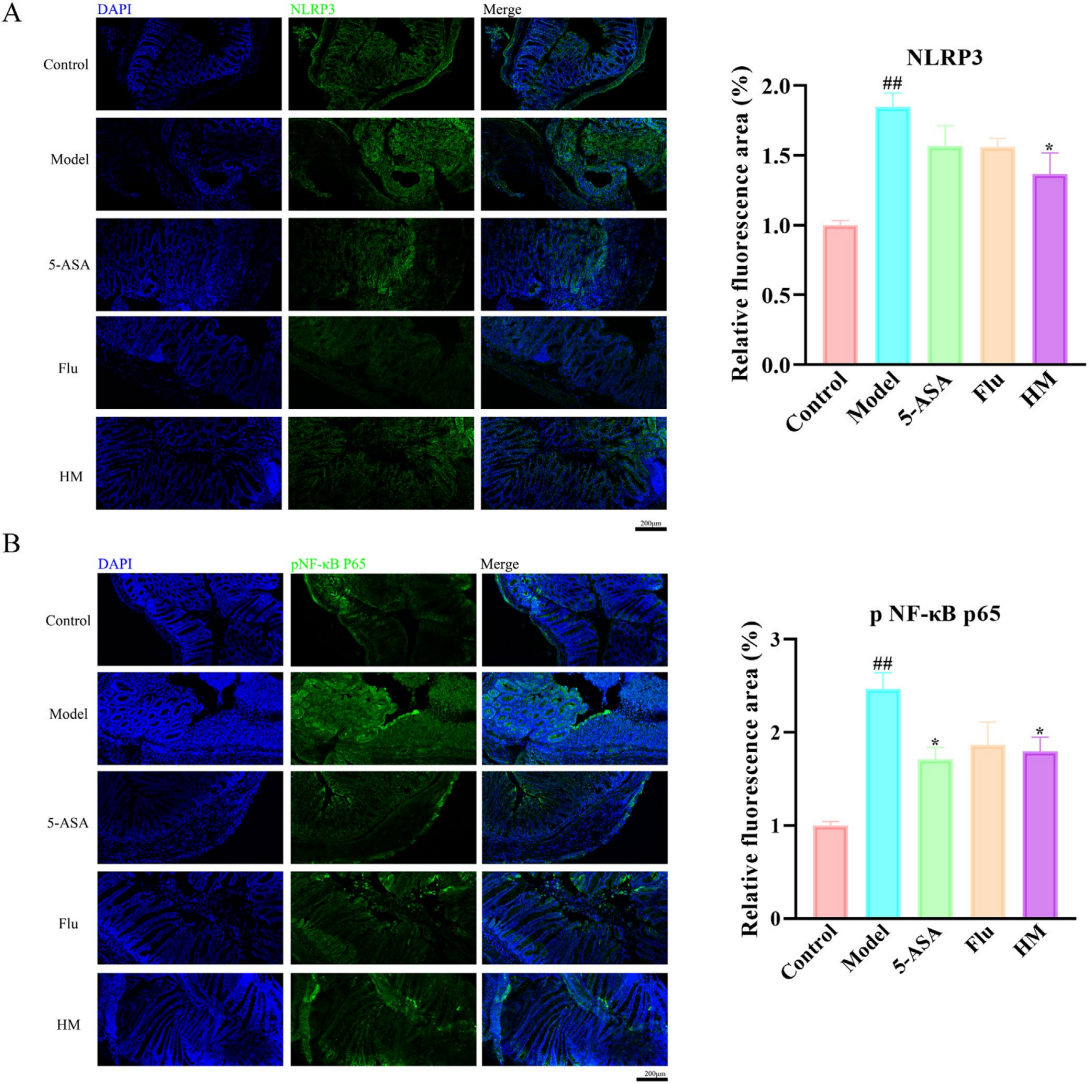
Effect of seahorses on DSS‐induced inflammation in mouse colon tissue (*n* = 8). (A) Immunofluorescence staining for NLRP3 in colonic sections of mice. (B) Immunofluorescence staining for NF‐κB p65 in colonic sections. Scale bars are set at 200 μm. #*p* < 0.05 and ##*p* < 0.01 vs. control group; *p < 0.05 and **p < 0.01 vs. DSS‐induced model group.

Building upon our observation that seahorse extract attenuates intestinal barrier dysfunction in DSS‐induced colitis, we examined its effects on key inflammatory signaling pathways (Figure 4). Our analysis focused on the pNF‐κB and NLRP3 pathways, which play critical roles in inflammatory responses. DSS treatment markedly upregulated both pNF‐κB and NLRP3 expression in colonic tissues, indicating the activation of inflammatory pathways. However, seahorse extract treatment significantly suppressed this activation, with substantial reductions in both pNF‐κB (a master regulator of inflammation) and NLRP3 (a core inflammasome component) levels compared to DSS‐treated controls.

### 3.5 Seahorse Inhibited the Neuroinflammation in the Hippocampal CA1 Region of DSS‐Induced Mice

**FIGURE 5:**
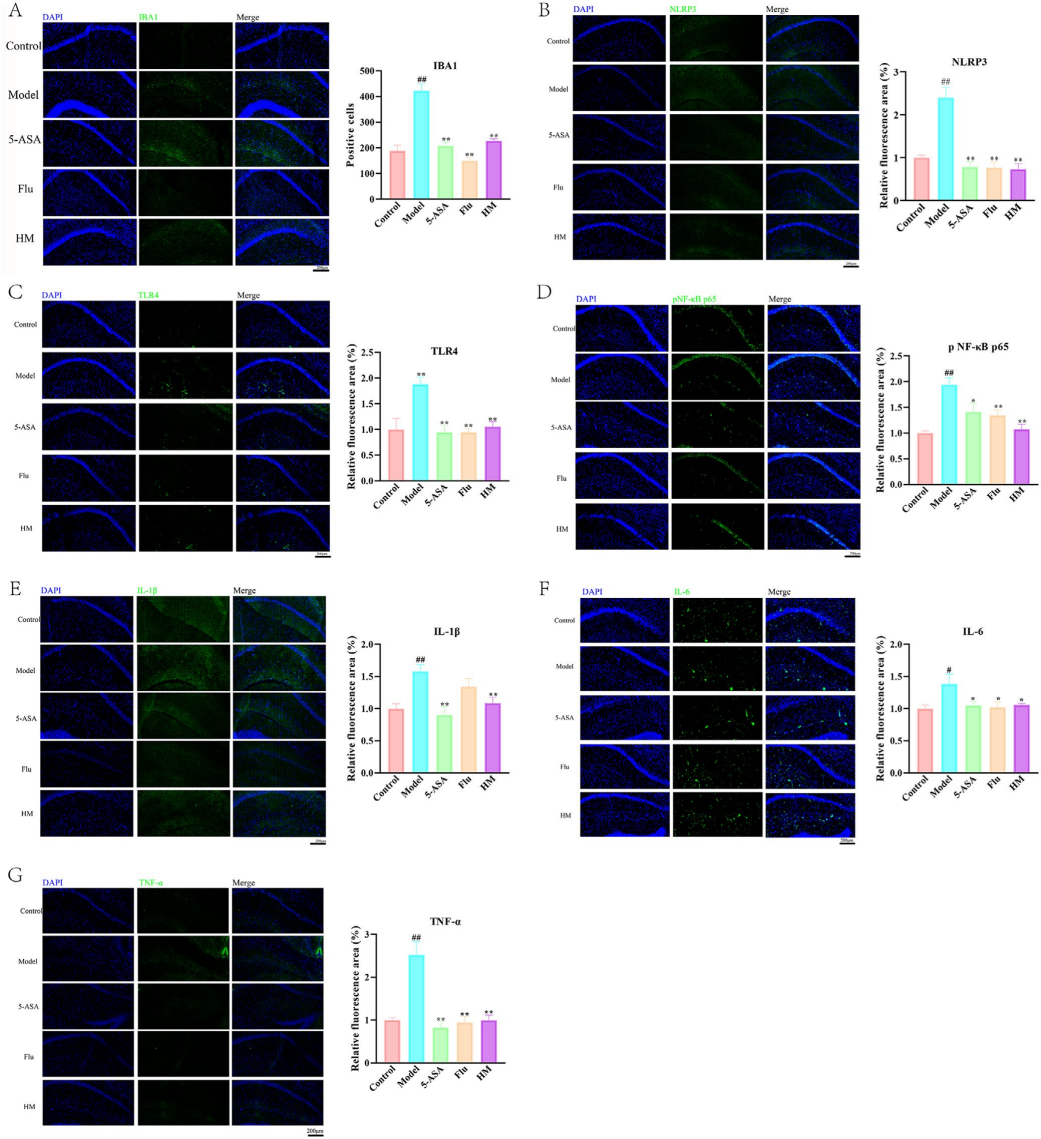
Effect of seahorses on DSS‐induced neuroinflammation in the hippocampal CA1 region (*n* = 6). (A) Quantitative and morphological analysis of microglia in the whole hippocampus of the mouse hippocampus. (B) Immunofluorescence staining for NLRP3. (C) Immunofluorescence staining for TLR4. (D) Immunofluorescence staining for phosphorylated NF‐κB. Immunofluorescence staining for proinflammatory cytokines (E) IL‐1β, (F) IL‐6, and (G) TNF‐α. Scale bar = 200 μm. #*p* < 0.05 and ##*p* < 0.01 vs. control group; *p < 0.05 and **p < 0.01 vs. DSS‐induced model group.

Given the established link between peripheral inflammation and central neuroinflammation in depression pathogenesis, we examined the effects of seahorse on neuroinflammatory markers in the hippocampal CA1 region, a critical area for cognitive function. As shown in Figure 5, seahorse treatment significantly reduced Iba1‐positive microglia in the CA1 region, indicating suppressed microglial activation—a key driver of neuroinflammatory damage. Furthermore, seahorse extract downregulated multiple inflammatory mediators including TLR4, pNF‐κB, and NLRP3, demonstrating broad anti‐neuroinflammatory effects. The extract also reduced levels of pro‐inflammatory cytokines predominantly released by activated microglia. These findings collectively demonstrated the ability of seahorse to attenuate DSS‐induced neuroinflammation in this clinically relevant brain region.

### 3.6 Seahorse Improved Neurotrophic Function in the Hippocampal CA1 Region of DSS‐Induced Mice

**FIGURE 6:**
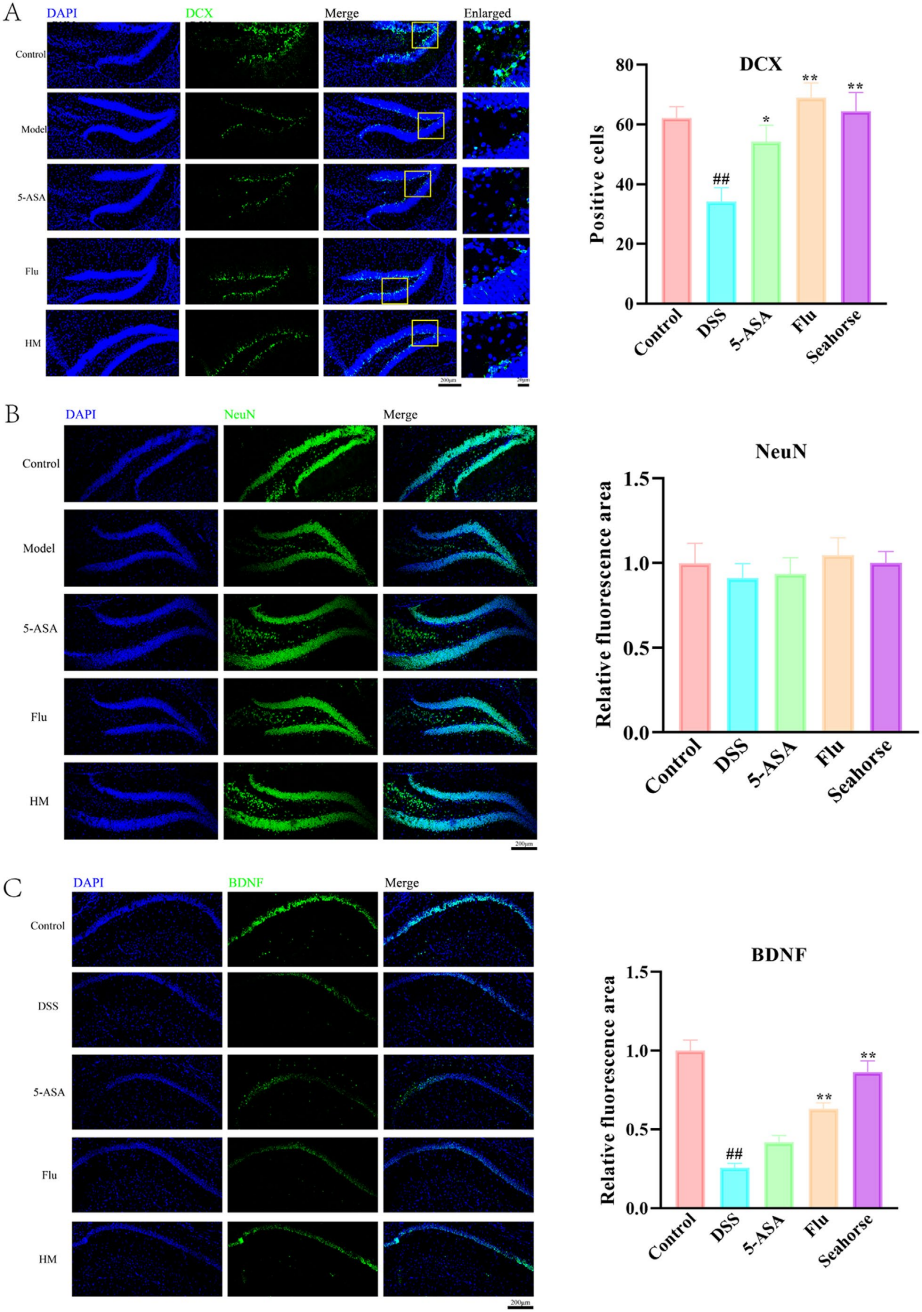
Effect of seahorses on the hippocampal neurotrophic function in hippocampal DG and CA1 region (*n* = 6). (A) Immunofluorescence staining for DCX in the DG region. (B) Immunofluorescence staining for NeuN in the DG region of the hippocampus. (C) Immunofluorescence staining for BDNF in the CA1 region. Scale bar = 200 μm. #*p* < 0.05 and ##*p* < 0.01 vs. control group; *p < 0.05 and **p < 0.01 vs. DSS‐induced model group.

Our analysis of hippocampal neuroplasticity revealed region‐specific effects of seahorse treatment (Figure 6). In the dentate gyrus (DG), seahorse extract significantly increased DCX+ cell numbers (Figure 6A), indicating stimulated neurogenesis—a critical process for cognitive and emotional regulation. Interestingly, neither DSS nor seahorse treatment altered total neuronal counts in the CA1 region (Figure 6B), suggesting preserved neuronal populations. Importantly, seahorse extract counteracted the DSS‐induced reduction of BDNF in CA1 (Figure 6C), restoring levels of this crucial neurotrophin that supported neuronal survival and synaptic plasticity (Pisani et al. 2023; Wu and Zhang 2023).

### 3.7 Seahorse Inhibited Hippocampal Ferroptosis Related Proteins in DSS‐Induced Mice

**FIGURE 7:**
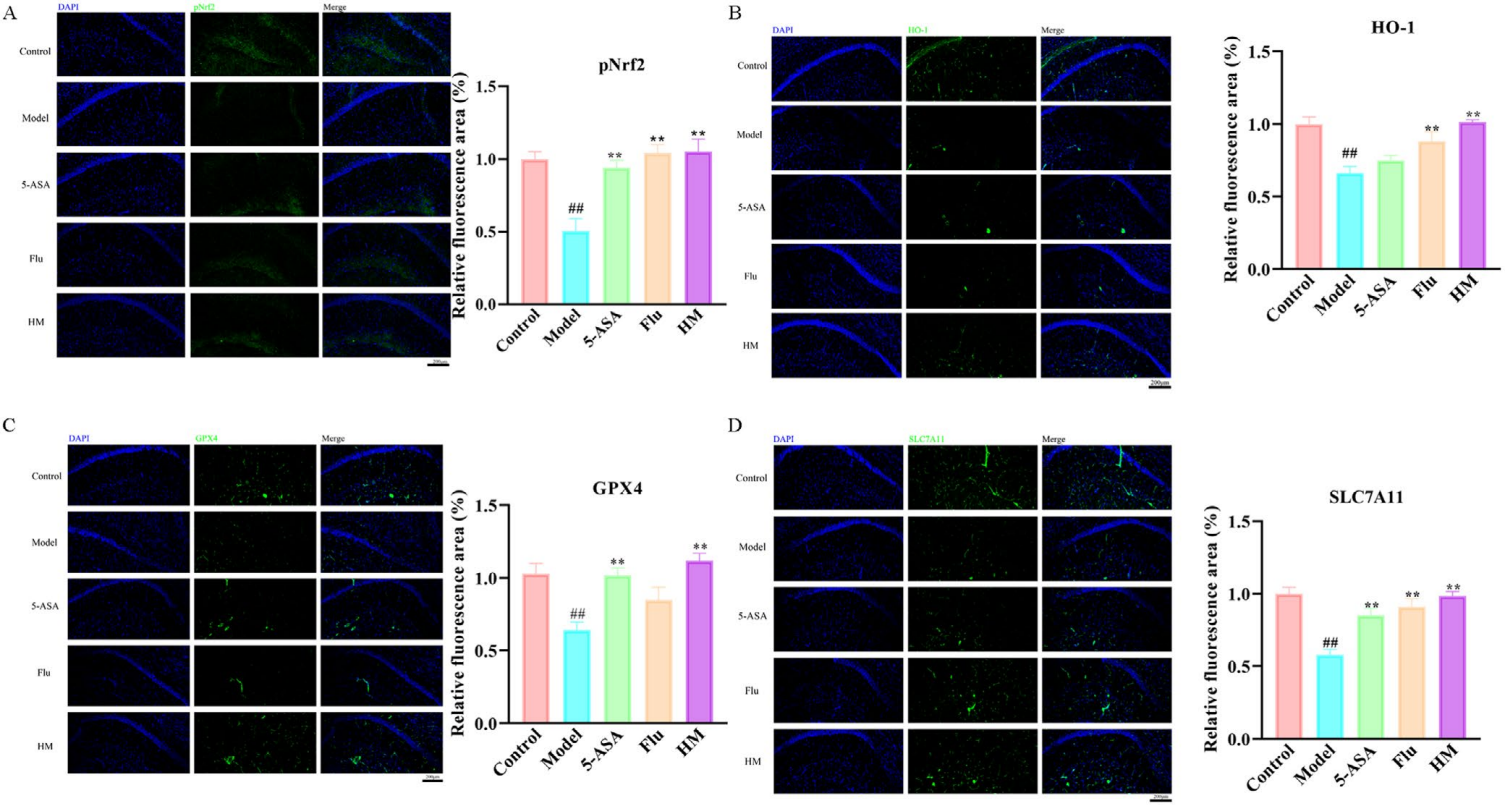
Effect of seahorses on DSS‐induced ferroptosis in hippocampal CA1 region (*n* = 4). (A) Immunofluorescence staining for pNrf2 in the CA1 region. (B) Immunofluorescence staining for HO‐1 in the CA1 region. (C) Immunofluorescence staining for GPX4 in the CA1 region. (D) Immunofluorescence staining for SLC7A11 in the CA1 region. Scale bar = 200 μm. #*p* < 0.05 and ##*p* < 0.01 vs. control group; *p < 0.05 and **p < 0.01 vs. DSS‐induced model group.

Building on the established role of BDNF in activating the Nrf2‐mediated antioxidative signaling pathway, a key mechanism counteracting ferroptosis, we assessed ferroptosis‐related protein expression in the hippocampal CA1 region. Our results (Figure 7) demonstrate that seahorse significantly upregulated pNrf2, HO‐1, GPX4, and SLC7A11 in this region. This coordinated upregulation indicated the potent modulation of the hippocampal antioxidative defense system, with pNrf2 activation driving expression of cytoprotective HO‐1, whereas elevated GPX4 (the key ferroptosis inhibitor) and SLC7A11 (the cystine/glutamate antiporter essential for glutathione synthesis) collectively protected against lipid peroxidation and ferroptotic cell death in DSS‐treated mice.

### 3.8 Seahorse Improved the Cellular Ultrastructure of Hippocampal Neurons in DSS‐Induced Mice

**FIGURE 8:**
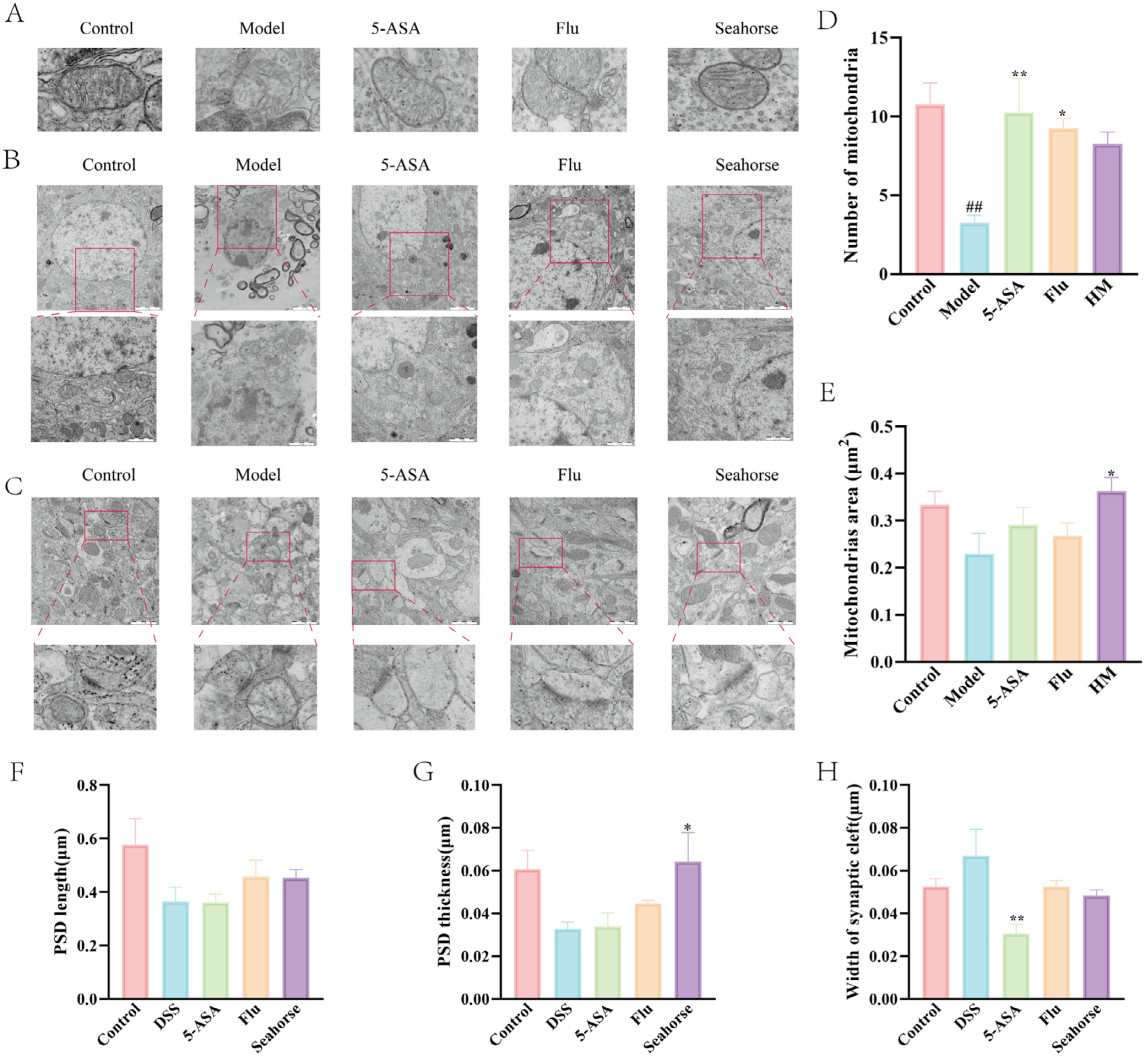
Effect of seahorses on the cellular ultrastructure of DSS‐induced mouse hippocampal CA1 neurons (*n* = 4). (A) TEM images showing ultrastructural alterations in hippocampal mitochondria of mice. This panel highlights mitochondrial damage associated with DSS‐induced stress. Scale bar = 2 μm. (B) TEM analysis depicting the density and size of mitochondria surrounding the nuclei in the hippocampal region. These images provide quantitative insights into mitochondrial health and morphology. Scale bars = 2 μm (overview) and 1 μm (enlarged view). (C) Detailed TEM images of synaptic morphology within the mouse hippocampus, illustrating the structural integrity of synapses. Scale bar = 1 μm. (D) The histogram of mitochondria number. (E) The histogram of mitochondrial size. (F) The histogram of PSD length. (G) The histogram of PSD thickness. (H) The histogram of width of synaptic cleft. (I) Immunofluorescence staining for AIF in the CA1 region of the mouse hippocampus, indicating the extent of ferroptosis‐induced neuronal apoptosis. Scale bar = 200 μm. Statistical significance is denoted as follows: #*p* < 0.05 and ##*p* < 0.01 when compared to the control group; *p < 0.05 and **p < 0.01 in comparison to the DSS‐induced model group.

Mitochondria, vital organelles for cellular energy metabolism, are the primary sites of ATP production. As delineated in Figure 8A–C, mitochondria in the control group exhibited well‐preserved ultrastructure, characterized by intact inner and outer membranes and clearly defined mitochondrial cristae. Contrastingly, in the DSS‐treated group, mitochondrial atrophy was evident, coupled with a marked absence of mitochondrial cristae. Furthermore, quantitative analysis revealed that, at equivalent magnification, the mitochondria surrounding the nuclei in the DSS group were significantly reduced in both size and number compared to those in the control group. Notably, seahorse extract treatment resulted in a substantial increase in mitochondrial numbers, enlargement of mitochondrial size, and restoration of mitochondrial cristae, relative to the DSS group. These findings suggest that seahorse extract can reverse the mitochondrial damage induced by DSS treatment. Additionally, we investigated the impact of seahorse extract on hippocampal synaptic plasticity in the DSS‐induced depression model, as depicted in Figure 8C,D. Morphological assessments revealed that, compared to the control group, DSS‐treated mice exhibited significant synaptic alterations, including shortened postsynaptic membrane length, decreased postsynaptic membrane thickness, and an expanded synaptic gap. Remarkably, seahorse treatment effectively reversed these DSS‐induced synaptic morphological changes.

## 4 Discussion

The present study investigated the antidepressant‐like effects of seahorse extract and its modulation of both ferroptosis and inflammation in a DSS‐induced depression model in mice. Our findings demonstrate that seahorse extract alleviates depressive‐like behaviors while regulating hippocampal molecular pathways involved in inflammation (through TLR4/NF‐κB/NLRP3 suppression) and ferroptosis (via pNrf2/HO‐1/GPX4 upregulation). These results highlight the therapeutic potential of seahorse extract for depression through novel mechanisms. Unlike previous studies that focused only on the antioxidative or anti‐inflammatory effects of seahorse (Kang et al. 2017), our work reveals its unique ability to simultaneously target both neuroinflammation and ferroptosis. Although Elbandy (2022) reviewed the anti‐neuroinflammatory potential of marine compounds generally (Elbandy 2022), we provided direct evidence that seahorse preserved mitochondrial integrity and synaptic plasticity, a novel finding in this field.

Neuroinflammation has emerged as a critical factor in depression pathophysiology, characterized by microglial activation and elevated pro‐inflammatory cytokine levels that exacerbate depressive symptoms (Wu and Zhang 2023). Our findings reveal that seahorse extract significantly reduced Iba1‐positive microglia in the hippocampal CA1 region, demonstrating potent anti‐neuroinflammatory activity. As the primary immune cells in the brain, activated microglia drive depression pathology through pro‐inflammatory cytokine release that sustains neuroinflammation and neuronal dysfunction (Zhu et al. 2023). Current antidepressant strategies frequently target microglial activation to restore neurochemical balance and alleviate symptoms (Wang et al. 2022). Seahorse extract showed comprehensive anti‐neuroinflammatory effects by suppressing key mediators including TLR4, pNF‐κB, and NLRP3 in the CA1 region, whereas also reducing microglial cytokine release. These findings collectively established the ability of seahorse to modulate critical neuroinflammatory pathways in depression.

Neurogenesis plays a critical role in depression pathophysiology, with impaired hippocampal neurogenesis strongly associated with depressive symptoms (Kim and Park 2021). Like conventional antidepressants that promote neurogenesis and upregulate BDNF to enhance neuronal growth and synaptic plasticity (Esalatmanesh et al. 2023), seahorse extract significantly increased DCX+ cells in the dentate gyrus (indicating enhanced neurogenesis) and elevated BDNF levels in the CA1 region. These findings demonstrated that seahorse extract shared key neurogenic and neurotrophic mechanisms with established antidepressants, supporting its potential therapeutic benefits for depression.

Recent studies have established ferroptosis—an iron‐dependent cell death pathway driven by lipid peroxidation—as a significant contributor to depression pathogenesis (Du et al. 2023). Characterized by the accumulation of lipid peroxides, ferroptosis is increasingly being recognized for its role in neuronal damage and the exacerbation of depressive disorders. The modulation of ferroptosis‐related pathways, such as the enhancement of antioxidative responses, is emerging as a novel therapeutic approach in reversing depression‐like behaviors. In light of these advancements, our study explored the effects of seahorse extract on key proteins involved in the ferroptosis pathway within the hippocampal CA1 region, an area crucial for emotional regulation and cognitive function. Notably, the administration of seahorse extract resulted in a significant upregulation of pNrf2, a master regulator of the cellular antioxidant response. Moreover, the expression of HO‐1, GPX4, and SLC7A11 was markedly increased following seahorse extract treatment. HO‐1 plays a typical role in cellular defense mechanisms against oxidative stress (Chiang et al. 2021), whereas GPX4 is a key enzyme in the detoxification of lipid peroxides, preventing the lethal accumulation of these peroxides that drive ferroptosis (Forcina and Dixon 2019). SLC7A11, a component of the xCT antiporter that regulates the cellular uptake of cystine for glutathione synthesis, is crucial for maintaining the redox balance and preventing ferroptosis (Koppula et al. 2018). The enhancement of these proteins by seahorse extract points to a multi‐faceted protective mechanism against ferroptosis, potentially contributing to the therapeutic effects observed in the DSS‐induced depression model. Our study reveals that the antidepressant effects are mediated through robust activation of the pNrf2/HO‐1 axis, a critical pathway counteracting hippocampal ferroptosis. The upregulation of pNrf2 and its downstream targets HO‐1 and GPX4 demonstrates the unique ability of seahorse to simultaneously enhance cellular antioxidant defenses (via HO‐1‐mediated heme degradation) and directly inhibit lipid peroxidation (via GPX4)—a dual mechanism distinct from conventional antidepressants. This is particularly relevant to depression pathophysiology, as Nrf2 dysfunction exacerbates oxidative stress‐induced neuronal atrophy, while GPX4 depletion accelerates ferroptotic death in mood‐regulating circuits (Zhou et al. 2025). Our findings thus identified seahorse as a novel modulator of both ferroptosis and neuroinflammation in depression, with particular relevance for hippocampal protection.

Our findings demonstrated that seahorse extract significantly preserved mitochondrial and synaptic integrity in the DSS‐induced depression model, highlighting its anti‐ferroptotic potential. In DSS‐treated mice, we observed characteristic ferroptosis‐related mitochondrial damage including atrophy, cristae loss, and reduced mitochondrial number/size—all indicators of iron‐dependent oxidative stress (Sun et al. 2023). Seahorse extract treatment effectively reversed these abnormalities, restoring mitochondrial ultrastructure (cristae morphology, number, and dimensions) to near‐normal levels. Crucially, the extract also ameliorated DSS‐induced synaptic pathology, normalizing postsynaptic density dimensions (length and thickness) and synaptic cleft width. These coordinated improvements in both mitochondrial health and synaptic ultrastructure strongly suggested that seahorse extract exerted its neuroprotective effects by mitigating ferroptotic processes. Given the established link between ferroptosis and depression‐related neural circuit dysfunction, our results positioned seahorse extract as a promising therapeutic candidate that targeted both mitochondrial integrity and synaptic connectivity in depressive disorders.

Our HPLC analysis identified key amino acids with established roles in inflammation and ferroptosis regulation. Previous study has shown that glycine pretreatment prevented LPS‐induced dysfunctions by inhibiting NLRP3 inflammasome and activating Nrf2 signaling (Zhang et al. 2020), which with our observed reductions in IL‐1β and NLRP3 levels, and increase in pNrf2 levels. In addition to amino acids, seahorse extract also contains bioactive fatty acids that contribute to its mechanistic effects (Feng et al. 2024). Omega‐3 polyunsaturated fatty acids (PUFAs), such as EPA and DHA, commonly found in marine organisms, are potent regulators of both inflammation and ferroptosis. PUFAs inhibited NLRP3 inflammasome activation by reducing mitochondrial ROS and preventing ASC speck formation (Yan et al. 2013). PUFAs also improved the antioxidant defense via an Nrf2‐dependent mechanism (Zgorzynska et al. 2017). Moreover, exogenous fatty acids could induce a ferroptosis‐resistant cell state by suppressing the accumulation of lipid peroxides and decreasing levels of oxidizable polyunsaturated fatty acids.

This study establishes seahorse as a promising functional food for managing gut‐brain axis disorders. Future investigations should focus on optimizing culinary preparation methods to preserve bioactive components, characterizing nutrient‐nutrient interactions in dietary contexts, and conducting clinical trials to evaluate translational potential. Importantly, conservation of seahorse populations must accompany these research efforts.

Although this study identifies the therapeutic potential of seahorse, several limitations warrant discussion. First, although HPLC analysis identified 17 amino acids (including glycine and glutamate), their individual contributions require further validation. Existing evidence suggests glycine acts as an NLRP3 inhibitor (Zhang et al. 2020), whereas glutamate modulates NF‐κB signaling (Meffert et al. 2003), potentially mediating the observed anti‐inflammatory effects. Second, the anti‐ferroptotic effects may come from two mechanisms. Glycine supports glutathione synthesis, which maintains GPX4 activity. Hydroxyproline chelates iron, reducing oxidative stress. However, other components like trace elements or lipids may also contribute synergistically. These potential interactions need further study from the following aspects: bioactivity‐guided fractionation to isolate active amino acid combinations, quantification of iron metabolism markers (ferritin, transferrin) to delineate mechanism, and comparative analysis with other neuroprotective foods to identify unique nutritional profiles.

This study demonstrated that seahorse extract exerted comprehensive antidepressant effects through three interconnected mechanisms: (1) attenuating neuroinflammation via reduced microglial activation and suppressed TLR4/NF‐κB/NLRP3 signaling in hippocampal CA1, (2) enhancing neurogenesis as shown by increased DCX+ cells and BDNF levels, and (3) inhibiting ferroptosis through preserved mitochondrial ultrastructure and synaptic integrity. By simultaneously targeting these pathological hallmarks of depression—neuroinflammation, impaired neuroplasticity, and oxidative stress—seahorse extract presented a novel, multi‐target therapeutic strategy. Our findings supported further investigation of seahorse‐based interventions for depression, particularly in cases involving gut‐brain axis dysfunction.

## Author Contributions

**Pei‐Lu Chen:** formal analysis (equal), investigation (equal), methodology (equal), writing – original draft (equal). **Ming Li:** investigation (equal). **Xin‐Yu Wang:** investigation (equal). **Xian‐Zhu Qiu:** formal analysis (equal). **Feng‐Yan Qiu:** formal analysis (equal). **Le‐Yun Zheng:** formal analysis (equal). **Jia‐Yuan Zhang:** conceptualization (equal), investigation (equal). **Li‐Tao Yi:** conceptualization (equal), supervision (equal), writing – review and editing (equal). **Guang‐Hui Xu:** conceptualization (equal), funding acquisition (equal), project administration (equal), supervision (equal), writing – review and editing (equal).

## Ethics Statement

All experimental procedures received prior approval from the Huaqiao University Institutional Animal Care and Use Committee, approval no. A2022043, and were conducted in strict accordance with guidelines set by the China Council on Animal Care.

## Conflicts of Interest

The authors declare no conflicts of interest.

## Data Availability

The data that support the findings of this study are available from the corresponding author upon reasonable request.

## References

[fsn370482-bib-0001] Chan, V. K. Y. , M. Y. M. Leung , S. S. M. Chan , et al. 2024. “Projecting the 10‐Year Costs of Care and Mortality Burden of Depression Until 2032: A Markov Modelling Study Developed From Real‐World Data.” Lancet Regional Health – Western Pacific 45: 101026. 10.1016/j.lanwpc.2024.101026. 38352243 PMC10862399

[fsn370482-bib-0002] Chassaing, B. , J. D. Aitken , M. Malleshappa , and M. Vijay‐Kumar . 2014. “Dextran Sulfate Sodium (DSS)‐Induced Colitis in Mice.” Current Protocols in Immunology 104: 15.25.11–15.25.14. 10.1002/0471142735.im1525s104. PMC3980572 24510619

[fsn370482-bib-0003] Chiang, S. K. , S. E. Chen , and L. C. Chang . 2021. “The Role of HO‐1 and Its Crosstalk With Oxidative Stress in Cancer Cell Survival.” Cells 10, no. 9: 2401. 34572050 10.3390/cells10092401 PMC8471703

[fsn370482-bib-0004] Chu, J. , J. Li , L. Sun , and J. Wei . 2023. “The Role of Cellular Defense Systems of Ferroptosis in Parkinson's Disease and Alzheimer's Disease.” International Journal of Molecular Sciences 24, no. 18: 14108. 37762411 10.3390/ijms241814108 PMC10531775

[fsn370482-bib-0005] Clapp, M. , N. Aurora , L. Herrera , M. Bhatia , E. Wilen , and S. Wakefield . 2017. “Gut Microbiota's Effect on Mental Health: The Gut‐Brain Axis.” Clinics and Practice 7, no. 4: 987. 10.4081/cp.2017.987. 29071061 PMC5641835

[fsn370482-bib-0006] Cui, J. J. , Z. Y. Huang , Y. H. Xie , et al. 2023. “Gut Microbiota Mediated Inflammation, Neuroendocrine and Neurotrophic Functions Involved in the Antidepressant‐Like Effects of Diosgenin in Chronic Restraint Stress.” Journal of Affective Disorders 321: 242–252. 10.1016/j.jad.2022.10.045. 36349650

[fsn370482-bib-0007] Dempsey, E. , A. Abautret‐Daly , N. G. Docherty , C. Medina , and A. Harkin . 2019. “Persistent Central Inflammation and Region Specific Cellular Activation Accompany Depression‐ and Anxiety‐Like Behaviours During the Resolution Phase of Experimental Colitis.” Brain, Behavior, and Immunity 80: 616–632. 10.1016/j.bbi.2019.05.007. 31063848

[fsn370482-bib-0008] Du, L. , Y. Wu , Z. Fan , et al. 2023. “The Role of Ferroptosis in Nervous System Disorders.” Journal of Integrative Neuroscience 22, no. 1: 19. 10.31083/j.jin2201019. 36722234

[fsn370482-bib-0009] Elbandy, M. 2022. “Anti‐Inflammatory Effects of Marine Bioactive Compounds and Their Potential as Functional Food Ingredients in the Prevention and Treatment of Neuroinflammatory Disorders.” Molecules 28, no. 1: 2. 10.3390/molecules28010002. 36615197 PMC9822486

[fsn370482-bib-0010] Esalatmanesh, S. , L. Kashani , and S. Akhondzadeh . 2023. “Effects of Antidepressant Medication on Brain‐Derived Neurotrophic Factor Concentration and Neuroplasticity in Depression: A Review of Preclinical and Clinical Studies.” Avicenna Journal of Medical Biotechnology 15, no. 3: 129–138. 37538241 10.18502/ajmb.v15i3.12922 PMC10634295

[fsn370482-bib-0011] Feng, B. Y. , H. Zhang , D. Y. Zhang , et al. 2024. “Comprehensive Biochemical Analysis and Nutritional Evaluation of Fatty Acid and Amino Acid Profiles in Eight Seahorse Species (*Hippocampus* spp.).” Heliyon 10, no. 12: e33220. 10.1016/j.heliyon.2024.e33220. 39021916 PMC11252734

[fsn370482-bib-0012] Forcina, G. C. , and S. J. Dixon . 2019. “GPX4 at the Crossroads of Lipid Homeostasis and Ferroptosis.” Proteomics 19, no. 18: e1800311. 10.1002/pmic.201800311. 30888116

[fsn370482-bib-0013] Kang, N. , S.‐Y. Kim , S. Rho , J.‐Y. Ko , and Y.‐J. Jeon . 2017. “Anti‐Fatigue Activity of a Mixture of Seahorse ( *Hippocampus abdominalis* ) Hydrolysate and Red Ginseng.” Fisheries and Aquatic Sciences 20, no. 1: 3. 10.1186/s41240-017-0048-x.

[fsn370482-bib-0014] Kim, I. B. , and S. C. Park . 2021. “The Entorhinal Cortex and Adult Neurogenesis in Major Depression.” International Journal of Molecular Sciences 22, no. 21: 11725. 34769155 10.3390/ijms222111725 PMC8583901

[fsn370482-bib-0015] Koppula, P. , Y. Zhang , L. Zhuang , and B. Gan . 2018. “Amino Acid Transporter SLC7A11/xCT at the Crossroads of Regulating Redox Homeostasis and Nutrient Dependency of Cancer.” Cancer Communications 38, no. 1: 12. 29764521 10.1186/s40880-018-0288-x PMC5993148

[fsn370482-bib-0016] Ma, Y. , T. Liu , X. Li , et al. 2022. “Estrogen Receptor Beta Deficiency Impairs Gut Microbiota: A Possible Mechanism of IBD‐Induced Anxiety‐Like Behavior.” Microbiome 10, no. 1: 160. 10.1186/s40168-022-01356-2. 36175956 PMC9520828

[fsn370482-bib-0017] Meffert, M. K. , J. M. Chang , B. J. Wiltgen , M. S. Fanselow , and D. Baltimore . 2003. “NF‐Kappa B Functions in Synaptic Signaling and Behavior.” Nature Neuroscience 6, no. 10: 1072–1078. 10.1038/nn1110. 12947408

[fsn370482-bib-0018] Ouabbou, S. , Y. He , K. Butler , and M. Tsuang . 2020. “Inflammation in Mental Disorders: Is the Microbiota the Missing Link?” Neuroscience Bulletin 36, no. 9: 1071–1084. 10.1007/s12264-020-00535-1. 32592144 PMC7475155

[fsn370482-bib-0019] Pisani, A. , F. Paciello , V. Del Vecchio , et al. 2023. “The Role of BDNF as a Biomarker in Cognitive and Sensory Neurodegeneration.” Journal of Personalized Medicine 13, no. 4: 652. 37109038 10.3390/jpm13040652 PMC10140880

[fsn370482-bib-0020] Puppala, E. R. , S. S. Yalamarthi , S. L. Aochenlar , et al. 2023. “Mesua Assamica (King&Prain) Kosterm. Bark Ethanolic Extract Attenuates Chronic Restraint Stress Aggravated DSS‐Induced Ulcerative Colitis in Mice via Inhibition of NF‐kappaB/STAT3 and Activation of HO‐1/Nrf2/SIRT1 Signaling Pathways.” Journal of Ethnopharmacology 301: 115765. 10.1016/j.jep.2022.115765. 36195303

[fsn370482-bib-0021] Réus, G. Z. , L. M. Manosso , J. Quevedo , and A. F. Carvalho . 2023. “Major Depressive Disorder as a Neuro‐Immune Disorder: Origin, Mechanisms, and Therapeutic Opportunities.” Neuroscience and Biobehavioral Reviews 155: 105425. 10.1016/j.neubiorev.2023.105425. 37852343

[fsn370482-bib-0022] Sun, K. , Y. Zhi , W. Ren , et al. 2023. “The Mitochondrial Regulation in Ferroptosis Signaling Pathway and Its Potential Strategies for Cancer.” Biomedicine & Pharmacotherapy 169: 115892. 10.1016/j.biopha.2023.115892. 37976895

[fsn370482-bib-0023] Wang, H. , Y. He , Z. Sun , et al. 2022. “Microglia in Depression: An Overview of Microglia in the Pathogenesis and Treatment of Depression.” Journal of Neuroinflammation 19, no. 1: 132. 35668399 10.1186/s12974-022-02492-0 PMC9168645

[fsn370482-bib-0024] Wang, J. , Z. Y. Yuan , X. Y. Wang , et al. 2024. “Anthocyanins‐Rich Cranberry Extract Attenuates DSS‐Induced IBD in an Intestinal Flora Independent Manner.” Current Research in Food Science 9: 100815. 10.1016/j.crfs.2024.100815. 39161885 PMC11332073

[fsn370482-bib-0025] Wu, A. , and J. Zhang . 2023. “Neuroinflammation, Memory, and Depression: New Approaches to Hippocampal Neurogenesis.” Journal of Neuroinflammation 20, no. 1: 283. 38012702 10.1186/s12974-023-02964-x PMC10683283

[fsn370482-bib-0026] Yan, Y. , W. Jiang , T. Spinetti , et al. 2013. “Omega‐3 Fatty Acids Prevent Inflammation and Metabolic Disorder Through Inhibition of NLRP3 Inflammasome Activation.” Immunity 38, no. 6: 1154–1163. 10.1016/j.immuni.2013.05.015. 23809162

[fsn370482-bib-0027] Yang, W. S. , and B. R. Stockwell . 2016. “Ferroptosis: Death by Lipid Peroxidation.” Trends in Cell Biology 26, no. 3: 165–176. 26653790 10.1016/j.tcb.2015.10.014 PMC4764384

[fsn370482-bib-0028] Yuan, Z.‐Y. , X.‐Y. Wang , J. Wang , et al. 2024. “Polygonatum Sibiricum Polysaccharides Attenuated Colitis via Regulating Gut Microbiota Mediated Colonic NLRP3/ASC/Caspase‐1/GSDMD Signaling Pathway.” Food Science and Human Wellness. 10.26599/FSHW.2024.9250291.

[fsn370482-bib-0029] Zgorzynska, E. , B. Dziedzic , A. Gorzkiewicz , et al. 2017. “Omega‐3 Polyunsaturated Fatty Acids Improve the Antioxidative Defense in Rat Astrocytes via an Nrf2‐Dependent Mechanism.” Pharmacological Reports 69, no. 5: 935–942. 10.1016/j.pharep.2017.04.009. 28662394

[fsn370482-bib-0030] Zhang, M. M. , M. X. Guo , Q. P. Zhang , et al. 2022. “IL‐1R/C3aR Signaling Regulates Synaptic Pruning in the Prefrontal Cortex of Depression.” Cell & Bioscience 12, no. 1: 90. 10.1186/s13578-022-00832-4. 35715851 PMC9205119

[fsn370482-bib-0031] Zhang, Y. , X. Ma , D. Jiang , et al. 2020. “Glycine Attenuates Lipopolysaccharide‐Induced Acute Lung Injury by Regulating NLRP3 Inflammasome and NRF2 Signaling.” Nutrients 12, no. 3: 611. 10.3390/nu12030611. 32110933 PMC7146254

[fsn370482-bib-0032] Zhang, Z. , L. Kong , M. Lv , et al. 2023. “PVA Enema Ameliorates DSS‐Induced Acute Colitis in Mice.” BMC Gastroenterology 23, no. 1: 368. 37904100 10.1186/s12876-023-03005-w PMC10617076

[fsn370482-bib-0033] Zhao, L. P. , J. Wu , W. Quan , et al. 2022. “DSS‐Induced Colitis Activates the Kynurenine Pathway in Serum and Brain by Affecting IDO‐1 and Gut Microbiota.” Frontiers in Immunology 13: 1089200. 10.3389/fimmu.2022.1089200. 36776388 PMC9908955

[fsn370482-bib-0034] Zhou, A. , H. Y. Feng , C. N. Fan , et al. 2025. “Asiaticoside Attenuates Chronic Restraint Stress‐Induced Hippocampal CA1 Neuronal Ferroptosis via Activating BDNF/Nrf2/GPX4 Signaling Pathway.” Drug Design, Development and Therapy 19: 793–810. 10.2147/DDDT.S509208. 39931216 PMC11808218

[fsn370482-bib-0035] Zhu, H. , A. Guan , J. Liu , L. Peng , Z. Zhang , and S. Wang . 2023. “Noteworthy Perspectives on Microglia in Neuropsychiatric Disorders.” Journal of Neuroinflammation 20, no. 1: 223. 37794488 10.1186/s12974-023-02901-y PMC10548593

